# Adverse childhood experiences are associated with vascular changes in adolescents that are risk factors for future cardiovascular disease

**DOI:** 10.1007/s00467-022-05853-2

**Published:** 2023-01-09

**Authors:** Cailin E. Kellum, Keri M. Kemp, Sylvie Mrug, Jennifer S. Pollock, Michael E. Seifert, Daniel I. Feig

**Affiliations:** 1 Cardio-Renal Physiology & Medicine, Division of Nephrology, Department of Medicine, University of Alabama at Birmingham, Birmingham, Alabama 35233 USA; 2 Division of Nephrology, Department of Pediatrics, University of Alabama at Birmingham, Birmingham, Alabama 35233 USA; 3 Department of Psychology, University of Alabama at Birmingham, Birmingham, Alabama 35233 USA

**Keywords:** Aortic augmentation index, Blood pressure, Early life stress, Adverse childhood experience, Adolescence

## Abstract

**Background:**

Adverse childhood experiences (ACEs), such as abuse, neglect, and household dysfunction, are associated with a higher risk of cardiovascular disease (CVD) and indicators of future CVD risk in adulthood, such as greater vascular stiffness. The impact of ACEs in adolescence is unclear, and understanding how ACEs relate to blood pressure (BP) and vascular function during early life is key for the development of prevention strategies to reduce CVD risk. We hypothesized that exposure to ACEs would be associated with changes in central hemodynamics such as increased vascular stiffness and higher BP during adolescence.

**Methods:**

This pilot study enrolled 86 adolescents recruited from the Children’s of Alabama. A validated ACE questionnaire was employed, and ACEs were modeled both as a continuous variable and a categorical variable (ACE ≥ 1 vs. ACE = 0). The primary outcomes used are considered to be indicators of future cardio-renal disease risk: aortic augmentation index normalized to 75 bpm (Alx75, a surrogate for vascular stiffness), carotid-femoral PWV (m/s), and ambulatory BP patterns.

**Results:**

Adolescents with ACE ≥ 1 had significantly higher Alx75 (ACE: 5.2% ± 2.2 compared to no ACE: − 1.4% ± 3.0; *p* = 0.043). PWV only reflected this trend when adjustments were made for the body mass index. Adolescents with ACEs showed no differences in ambulatory BP patterns during the 24-h, wake, or sleep periods compared to adolescents with no ACEs.

**Conclusions:**

ACEs were associated with higher AIx75 in adolescence, which is a risk factor for future CVD. Adolescence could present an opportunity for early detections/interventions to mitigate adverse cardiovascular outcomes in adulthood.

**Graphical abstract:**

**Figure:**
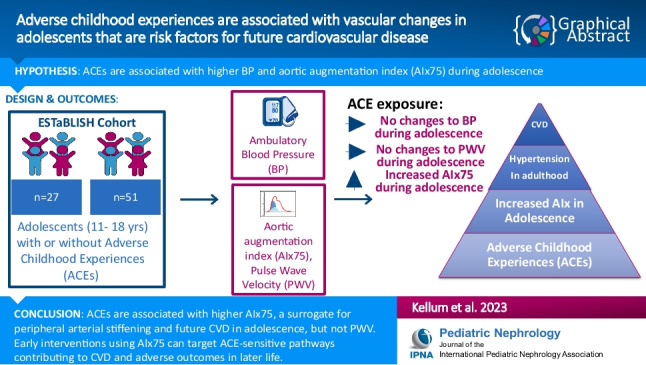
A higher resolution version of the Graphical abstract is available as Supplementary information

**Supplementary information:**

The online version contains supplementary material available at 10.1007/s00467-022-05853-2.

## Introduction

Adverse childhood experiences (ACEs) are traumatic events occurring before the age of eighteen years and include separation from parents, household dysfunction, and child maltreatment, such as neglect and verbal, physical and sexual abuse. The prevalence of ACEs is high in the USA; over 60% of US adults report experiencing at least one or more ACEs [1]. Since the foundational Kaiser Permanente study in 1995, which uncovered the link between ACEs and disease burden among adults, our understanding of the long-term impacts of ACEs has evolved. ACEs are now known to be a novel cardiovascular disease (CVD) risk factor in addition to increasing the likelihood of other CVD risk factors including diabetes, hypertension, and kidney diseases [2–4].

For example, a longitudinal study showed that individuals with ACEs have increased blood pressure (BP) trajectories compared to those without ACEs [4, 5]. However, to our knowledge, no study of ACEs and future CVD risk have utilized ambulatory BP monitoring, the gold standard assessment for abnormal BP. In addition, ACEs have been associated with higher pulse wave velocity (PWV, a surrogate for central vascular stiffness) in both adults and children [3, 6]. However, these prior studies of ACE and PWV have not focused on the adolescent, pediatric population nor assessed carotid-femoral PWV or pulse wave analysis metrics, such as aortic augmentation index (AIx) [6]. Because the links between ACEs and CVD risk factors have been studied primarily in adults, little is known about the relationships between ACEs and indicators for future CVD risk in adolescence.

To address these gaps from prior studies and better understand the association between ACEs and hemodynamic parameters in adolescence, we used non-invasive techniques to assess vascular stiffness including PWV and aortic augmentation index (Alx75, measure of the central arterial pressure enhanced by the wave reflection), which are surrogates for predicting future risk for hypertension, chronic kidney disease, and CVD [7–9]. In addition, we performed 24-h ambulatory BP monitoring (ABPM) to determine if the impact of ACEs on BP seen in adults can be detected as early as adolescence. ABPM has a clear benefit to reduce the burden of CVD risk early in life by reducing variability in readings and highlighting the distinction between types of hypertension [10, 11]. The ultimate goal of this and future studies is to identify the sub-population within those with ACE exposure who would benefit from the early intervention of intermediate risk factors as a means to prevent future hypertension, kidney, and cardiovascular disease [12].

## Methods

### Participants

We designed the Impact of Early Life STressors on the BLood Pressure and Vascular Function In AdoleScents and CHildren (ESTaBLISH) pilot study to test the hypothesis that adolescents with ACE exposure would have changes in central hemodynamics such as increased vascular stiffness and higher blood pressure, both of which are surrogates for overt cardiovascular (CV) disease. ESTaBLISH recruited male and female adolescents (age 11–18) presenting to Hypertension and General Pediatrics Clinics at Children’s of Alabama in Birmingham, AL, between June 2018 and January 2020. Exclusion criteria included previously diagnosed CV or kidney disease and chronic use of anti-hypertensive, anti-inflammatory, or stimulant ADHD medications. Patients previously diagnosed with hypertension in their records were excluded to avoid bias. The study was approved by the University of Alabama at Birmingham Institutional Review Board (IRB 300,001,423), and all recruitment and informed consent procedures involving human subjects adhered to guidelines set forth in the Declaration of Helsinki.

Participants were scheduled for a single visit to the research unit where they self-reported family history of hypertension and exposure to childhood adversity, followed by measurement of vascular function and body mass index (BMI, kg/m^2^) by trained nurses. Participants were then given instructions and an ABPM device to wear for 24 h after the visit. The self-reported race was categorized as white and non-white due to limitations in racial category size [non-white included American Indian or Alaska Native (*n* = 1), Asian (*n* = 6), Native Hawaiian or Other Pacific Islander (*n* = 0), African American (*n* = 39), and mixed race (*n* = 4)]. Only three individuals reported ethnicity of Hispanic or Latino, so we were limited to using only race in our analysis rather than race and ethnicity.

### Measures

#### Adverse childhood experiences questionnaire

ACE exposure was measured by self-report on the Adverse Childhood Experiences questionnaire [3]. The survey was completed after receiving written informed consent by a computer-assisted personal interview (CAPI) with a trained interviewer. This measure asks whether the youth ever experienced each of the 10 ACEs, including abuse (emotional, physical, sexual), neglect (emotional or physical), and household dysfunction (e.g., loss of a parent, exposure to substance abuse, mental illness, criminal household member, or domestic violence in the home). The number of endorsed experiences was summed, resulting in a score between 0 and 10 ACEs.

#### Vascular stiffness

Vascular stiffness was measured by both carotid-femoral PWV (m/s) and pulse wave analysis (PWA). PWV and PWA were measured using the SphygmoCor XCEL System (Atcor Medical, Sydney, Australia) using the average of 3 readings that passed internal quality control measures on the device. In addition, AIx75 and percent wave reflection from PWA were measured. AIx75 is calculated from augmentation pressure (AP), which is the difference between the primary outgoing wave and the reflected wave of the central arterial waveform, and pulse pressure (PP). The full formula used was AIx75 = AP/PP, indexed to a heart rate of 75 beats per minute to adjust for the effect of heart rate as suggested by previous studies [13].

#### Ambulatory blood pressure monitoring

Ambulatory blood pressure monitoring (ABPM) was measured with the SpaceLabs™ OnTrak device (Catalog 90,227, Spacelabs Healthcare, Snoqualmie, WA) using published guidelines [10]. The arm circumference was measured to determine the appropriate cuff size. Age, sex, height, and mean casual BP readings were recorded at the time of placement of the ABPM. ABPMs were programmed to measure BP every 20 min over a 22–26-h recording period. ABPM recordings with < 1 mmHg nighttime BP measurement value were excluded from the analysis. Readings included day, night, or 24-h diastolic blood pressure (DBP), systolic blood pressure (SBP), and mean arterial pressure (MAP). Participants recorded wake and sleep times in a diary that was returned to the research team with the ABPM device in a pre-paid shipping envelope. ABPM reports were interpreted by one of two pediatric nephrologists (D.I.F. or M.E.S.).

Casual, or clinic BP, was measured and categorized using a GE Healthcare Dinamap Pro-400 oscillometric device for a standardized protocol that followed recent clinical practice guidelines [11]. BP was measured according to the following parameters: (1) being seated at rest for at least 10 min, (2) taking four BP measurements, each 3–4 min apart, (3) using the right arm, and (4) averaging the second, third, and fourth readings.

### Statistical analysis

All statistical analyses were performed with SPSS version 27 and R-studio version 1.2.5033 [14]. The primary exposure was ACE scores, modeled as a continuous variable and also dichotomized as *No ACE* (ACE = 0) and *ACE* (ACE ≥ 1). ACE distribution can be seen in Supplementary Fig. 1. Consistent with prior research, dichotomized ACE scoring has been shown to be impactful; exposure to just one ACE is associated with elevated hypertension risk in young adults [15]. Differences in demographics, family history of hypertension, and BMI between the *No ACE* and *ACE* groups were assessed using Chi-squared tests for categorical variables and Wilcoxon signed-rank tests for continuous variables.

The pre-specified primary outcomes were mean ambulatory BP metrics, mean PWV, and mean AIx75, and they were compared with *ACE* and *No ACE* groups with Wilcoxon signed-rank tests. We also analyzed relationships between the number of ACE exposures and these CV metrics using unadjusted and adjusted linear regression models. Adjusted models included the covariates sex and BMI due to the preliminary bivariate analysis described above. For CV metrics that had a significant unadjusted association with ACE exposure, a secondary unadjusted linear regression analysis was conducted using the cumulative ordinal ACE score. All statistical analyses were performed at the 95% confidence interval with alpha ≤ 0.05, and standardized beta coefficients are reported for linear regressions. Distributions of BMI had non-normal curves determined by a Sharpiro-Wilk W Test (W = 0.89, *p* < 0.001); therefore, BMI was log-transformed in linear regression analysis.

An a priori power analysis was performed for PWV and ambulatory BP as part of the study design. With a sample size of at least 27 patients in each group, we could detect a 2 m/s absolute difference in PWV and a medium effect size of 0.5 for 24-h ambulatory BP between *No ACE* and *ACE* groups with 80% power, considering an alpha of 0.05.

## Results

### Cohort characteristics

The study enrolled 86 participants, of which 76 (88%) completed all study procedures and had complete data for inclusion in the final analysis. Race composition, age, and body mass index (BMI) of the cohort are described in Table 1. The median age of the cohort was 16 years (range 13–18 years). Overall, 51 (65%) participants reported at least one ACE exposure, consistent with prior studies in adults [1]. There were no significant differences in demographic characteristics, family history of hypertension, or BMI between the ACE (ACE ≥ 1) and No ACE (ACE = 0) groups (Table 1).

**Table 1:** Characteristics of the ESTaBLISH Cohort stratified by ACE exposure

	ACE = 0	ACE ≥ 1	*p*-value
*N*	27	51	
Age (years)	15 (13⎼18)	16 (13⎼18)	0.39
Sex			1.00
Male	14 (52%)	25 (49%)	
Female	13 (48%)	26 (51%)	
Race			0.49
White	11 (41%)	16 (31%)	
Non-white	15 (56%)	35 (69%)	
Not reported	1	0	
Family history of hypertension	21 (78%)	40 (78%)	0.63
Not reported	0	4	
Body mass index (kg/m^2^)	27.0 ± 1.9	27.0 ± 1.1	0.36

Values are presented as number of cases (percent prevalence), median years (range), or mean ± standard error. *P*-values are reported from bivariate analysis of ACE exposure and categorical variables using Chi-squared tests and continuous variables using Wilcoxon signed-rank tests

*ACE*, adverse childhood experiences

To determine which covariates should be included in adjusted models, the association of BP and vascular stiffness measurements was assessed with Wilcoxon signed-rank tests for binary demographic characteristics and with linear regression analysis for BMI. Sex was significantly correlated with mean SBP (*p* < 0.001), daytime SBP (*p* = 0.001), daytime MAP (*p* = 0.04), PWV (*p* = 0.003), and AIx75 (*p* < 0.001). BMI was significantly correlated with mean 24-h SBP (beta = 0.442, *R*^2^ = 0.185, *F*_1,76_ = 18.450, *p* < 0.001), daytime SBP (beta = 0.392, *R*^2^ = 0.143, *F*_1,76_ = 13.840, *p* < 0.001), nighttime SBP (beta = 0.452, *R*^2^ = 0.005, *F*_1,76_ = 19.570, *p* < 0.001), mean 24-h MAP (beta = 0.280, *R*^2^ = 0.066, *F*_1,76_ = 6.448, *p* = 0.013), daytime MAP (beta = 0.236, *R*^2^ = 0.0433, *F*_1,76_ = 4.489, *p* = 0.0374), nighttime MAP (beta = 0.282, *R*^2^ = 0.068, *F*_1,76_ = 6.588, *p* = 0.012), and PWV (beta = 0.338, *R*^2^ = 0.102, *F*_1,74_ = 4.18, *p* = 0.003). Height was significantly correlated with mean 24-h SBP (beta = 0.306, *R*^2^ = 0.119, *F*_1,76_ = 11.44, *p* = 0.001), daytime SBP (beta = 0.306, *R*^2^ = 0.0817, *F*_1,76_ = 7.848, *p* = 0.006), nighttime SBP (beta = 0.429, *R*^2^ = 0.173, *F*_1,76_ = 17.11, *p* < 0.001), nighttime MAP (beta = 0.302, *R*^2^ = 0.0782, *F*_1,76_ = 7.623, *p* = 0.007), and PWV (beta = 0.359, *R*^2^ = 0.1172, *F*_1,76_ = 10.95, *p* = 0.0014). Thus, the significant covariates for BP, PWV, and Alx75 were determined and used to build linear regression models.

### ACEs and measures of vascular function

**Fig. 1:**
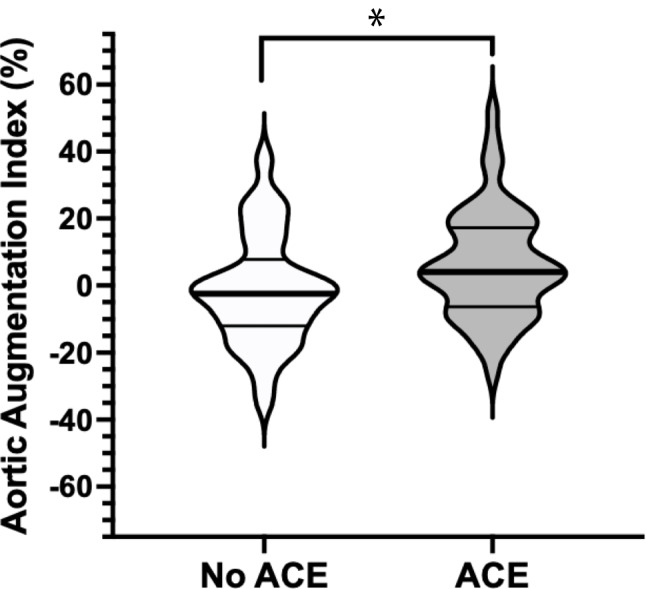
ACE exposure and AIx75 in adolescents. Adolescents with ≥ 1 ACE exposure have significantly higher AIx75 than those with no ACE exposure. Asterisk (*) denotes significance by a Mann–Whitney U test (*p* = 0.03) as well as by an unadjusted linear regression model (beta = 0.22, *p* = 0.043) shown in Table 3

Participants with ACEs had significantly higher AIx75 compared to those without ACEs (Table 2 and Fig. 1). In unadjusted linear regression models, the presence of ACEs was associated with higher AIx75 (beta = 0.22, *p* = 0.043), and this relationship was robust to adjustment for sex and BMI (beta = 0.21, *p* = 0.047) (Table 3). In unadjusted linear regression models where ACE was modeled as a continuous variable, increases in ACEs were associated with higher AIx75 (beta = 0.24, *p* = 0.03) (Supplementary Table 1). Carotid-femoral PWV, a direct measure of arterial stiffness, was positively associated with BMI, as previously reported in adolescents [8, 16, 17]. We found no differences in mean PWV between *ACE* and *No ACE* groups (Table 2). However, in adjusted linear regression models, we found a significant interaction of BMI and ACE with PWV (beta =  − 2.92, *p* = 0.01), where the effect of BMI on PWV was less positive in the ACE group compared to the No ACE group (Supplementary Table 2 and Supplementary Fig. 2). MAP was not a significant covariate of PWV (beta = 0.002, *p* = 0.98), and when models adjusted for MAP, the interaction of ACE and BMI on PWV remained significant (beta = 0.02, *p* = 0.031).

**Table 2:** Hemodynamic parameters ESTaBLISH cohort stratified by ACE exposure

	ACE = 0	ACE ≥ 1	*p*-value
PWV (m/s)	*n* = 26 5.3 ± 0.2	*n* = 50 5.4 ± 0.1	0.99
AIx75 (%)	*n* = 29 − 1.4 ± 3.0	*n* = 55 5.2 ± 2.2	0.03
SBP (mm Hg)	*n* = 27	*n* = 51	
24-h mean	110.9 ± 1.9	111.3 ± 1.3	0.89
Daytime mean	115.4 ± 2.0	115.3 ± 1.5	0.93
Nighttime mean	102.9 ± 2.3	103.8 ± 1.4	0.58
DBP (mm Hg)	*n* = 27	*n* = 51	
24-h mean	64.1 ± 0.9	65.9 ± 0.7	0.13
Daytime mean	68.6 ± 1.2	69.6 ± 0.8	0.45
Nighttime mean	56.1 ± 1.2	58.3 ± 0.8	0.08
MAP (mm Hg)	*n* = 27	*n* = 51	
24-h mean	110.9 ± 1.9	111.3 ± 1.3	0.40
Daytime mean	84.1 ± 1.3	84.7 ± 0.9	0.89
Nighttime mean	72.7 ± 1.1	74.9 ± 6.2	0.14
Heart rate (bpm)	*n* = 27 68.0	*n* = 51 68.5	0.91

Values are presented as number of cases (percent prevalence) or mean ± standard error. *P*-values are reported from bivariate analysis of ACE exposure and continuous hemodynamic variables using a Mann–Whitney U test

*ACE*, adverse childhood experiences; *PWV*, pulse wave velocity; *AIx*, augmentation index; *DPB*, diastolic blood pressure; *SBP*, systolic blood pressure; *MAP*, mean arterial pressure

**Table 3:** Unadjusted and adjusted effect of adverse childhood experience (ACE) on AIx

	Model 1 (unadjusted)		Model 2 (adjusted)	
Variable	*p*-value	β coef	*p*-value	β coef
ACE (0 vs. ≥ 1)	0.043	0.22	0.047	0.21
log(BMI)	-	-	0.06	0.20
Sex	-	-	0.40	0.01
ACE (0 vs. ≥ 1) * log(BMI)	-	-	-	-
Adjusted R^2^	0.04		0.06	
*F* statistic (*p*-value)	4.24 (0.04)		2.89 (0.04)	
Intercept (*p*-value)	− 1.42 (0.60)		38.97 (0.04)	

*AIx*, augmentation index; *BMI*, body mass index; *β coef*, standardized beta coefficient

### ACEs and ambulatory blood pressure.

The 24-h mean, daytime mean, and nighttime mean blood pressures in the *ACE* and *No ACE* groups are presented in Table 2. We found no differences in heart rate, day, night, or 24-h ambulatory BP metrics between the *ACE* and *No ACE* groups (Table 2). Unadjusted linear regression models found no associations between the *ACE* vs. *No ACE* groups and day, night, or 24-h ambulatory SBP, DBP, and MAP (Supplementary Table 3). The effect of ACE exposure on these hemodynamic parameters remained insignificant when adjusting for the covariates sex and BMI in linear regression models (Supplementary Table 3).

## Discussion

Prior studies focused on adults have established that ACE exposure amplifies the risk for hypertension and CVD [1, 4, 18], but opportunities for early intervention and mitigation of CVD risk are lacking. We found that adolescents who had experienced one or more ACEs had higher AIx75 compared to those with no ACE exposure, but showed no differences in casual or ambulatory BP. Our findings suggest that ACEs may be associated with early changes in vascular function in adolescents, prior to changes in casual or ambulatory BP that have been demonstrated in adulthood.

### ACEs and vascular stiffness in adolescents

Vascular stiffness is an early indicator of future CVD and hypertension [19–21]. Increased vascular stiffness in children as young as ten years has been proposed as a target for early intervention to mitigate future CVD risk, in conjunction with addressing other risk factors such as obesity, tobacco use, and low socioeconomic status [ 22–24]. Studies have shown increased vascular stiffness in adults who have experienced ACEs [3]. In children, the Niagara Longitudinal Heart Study found four or more ACEs were associated with a greater increase in vascular stiffness (assessed by PWV) over time from childhood into young adulthood independent of sex, systolic BP, body mass index, and physical activity [25]. Klassen et al. found that younger male children aged 10–14 years with ≥ 4 ACEs had greater PWV [6].

In the context of these previous studies, we assessed both PWV and AIx75 to more comprehensively assess the relationship between ACEs and vascular stiffness in adolescents. AIx75 has been suggested to be a more sensitive, reproducible marker of peripheral arterial stiffening in adolescents and is corrected for heart rate [26, 27]. AIx75 also measures central arterial pressure enhanced by wave reflection and has been associated with CVD risk factors in the overall population [28]. On the other hand, PWV is an indicator of central aortic stiffness, has been associated with hypertension-related CVD, and is used more often as a surrogate for future CVD risk in prior studies of ACEs [26, 27].

We found that individuals with one or more ACEs had higher AIx75 but not PWV. PWV is typically expected to correlate with AIx75 since the speed of the pulse wave is related to the amplitude of the reflected wave, but the literature suggests changes in AIx75 are more evident in younger subjects while PWV changes are more evident in older subjects [29]. Another potential explanation for the discordant findings with AIx75 and PWV is that changes in peripheral vascular stiffness may occur prior to changes in central vascular stiffness, such that exposure to a higher number of ACEs for a longer period of time may be required to see an association with increased PWV that was reported in previous studies [6, 25].

Additionally, although we found no association between ACEs and PWV alone, the effect of exposure to ≥ 1 ACEs on PWV was dependent on BMI. Specifically, BMI was more strongly related to higher PWV in those with no ACEs compared with ACEs. BMI is well documented to be associated with higher PWV in adolescence [16, 30–32]. Our results suggest that ACE exposure may decrease this relationship.

Although BMI was significantly correlated with PWV in our cohort which corroborates other studies [16, 30–32], BMI was not significantly correlated with Alx75 in our cohort. Alx75 has shown inconsistent associations with BMI with some studies finding a negative correlation in children and others finding a positive association in adolescents [8, 33, 34]. In the Klassen study, both exposure to ≥ 4 ACEs and BMI were significant predictors of vascular stiffness, consistent with our findings using a lower number of ACE exposures. Pierce et al. [8] found that obese and overweight African American youth have significantly higher PWV but no differences in AIx75. This suggests PWV may be more sensitive to vascular stiffness in children within a higher range of BMI [8]. Together, our results indicate that ACE-associated changes in hemodynamic function occur early in life and that increased AIx75 in adolescence may be one of the earliest detectable hemodynamic changes across a wide number of ACE exposures.

### ACEs and ambulatory BP in adolescents

Early identification and treatment of CVD risk factors are crucial to prevent irreversible target organ damage [35, 36]. Studies of cohorts in older adults have identified an association between exposure to ACEs and the risk of developing hypertension as an adult [4]; however, there are few data addressing adolescents [15, 37]. Previously, the Georgia Stress and Heart Study found in predictive models that the lifetime trajectory of SBP and DBP significantly increases with the number of ACEs after 30 years of age [5].

Similar to our findings, Gooding et al. evaluated a young adult cohort (24–32 years) and found that child maltreatment exposure was not associated with changes in office BP [38]. In a young adolescent cohort, Pretty et al. reported that in children with exposure to 4 or more ACEs, heart rate was significantly increased compared to those with fewer ACEs, but no differences were seen in office BP. Our study is strengthened by the use of ABPM which has not previously been completed in adolescents with and without ACEs. We found that ACE exposure was not linked with higher mean (24-h, daytime, nighttime) DBP, SBP, and MAP in adolescence. These findings, taken together with prior studies, suggest that overt hypertension, while associated with ACE exposure, could take several decades and/or additional stressors to manifest, while subtle intermediate risk factors such as altered Alx75 are detectable sooner after exposure. Early changes in peripheral vascular stiffness reflected by AIx75 could contribute to the subsequent increases in DBP and SBP seen in studies of adults that reported ACEs exposures as children.

### Limitations

Limitations of this study include the lack of information on the timing of ACEs experienced by the participants, which may be relevant to their effects [18, 39]. Due to the low prevalence of Hispanics in our cohort and low representations of certain races, we were limited in our analysis of ethnicity and race as covariates, but this deserves further study going forward. It is well known that racial and ethnic disparities exist in CV outcomes. Differential exposure to categories of ACEs has been linked to racial and ethnic groups that historically experience social and economic disadvantages [40]. Andrade and colleagues found that male Hispanic adolescents exposed to ≥ 4 ACEs had elevated casual DBP [41].

Additionally, the majority of individuals with ACEs in this cohort primarily experienced household dysfunction (59%) rather than sexual/physical abuse or neglect. While cumulative ACE exposure has been useful for understanding negative CV outcomes, the lack of specificity in abuse type may contribute to masking category-specific pathways to vascular dysfunction and hypertension [1]. For instance, maltreatment including verbal and physical assault has been linked to poorer mental health outcomes in adulthood in comparison to household dysfunction [42]. Few studies have examined the individual effects of abuse type on CV, and far fewer have focused on the type of adversity’s relative impact on future outcomes in comparison to one another.

We acknowledge that while this was designed as an initial study that was powered to detect moderate effect sizes between ACE groups, we were likely underpowered to detect more subtle differences in CV outcomes. Similarly, the pilot nature of this study did not permit us to split the cohort into a discovery and internal validation set. These considerations should be incorporated into future studies to test the association between ACE exposure and surrogates for CV risk.

### Conclusions

In summary, we found that ACE exposures were associated with early signs of vascular dysfunction reflected by increased AIx75 in adolescence. This is important since AIx75 is not routinely performed in clinical assessments of CV risk in adolescents, yet it may increase prior to changes in BP in adolescents exposed to ACEs. Overall, these results expand our current knowledge of the relationship between ACEs and hemodynamic parameters during adolescence and support the need for more research on ACE-related cardiovascular function in adolescence.

## Perspectives

Several physiological systems have been proposed to mediate the associations between ACEs and CVD in adulthood. It has been suggested that ACEs have prolonged programming effects on the humoral, nervous, and immune system responses to stressors and enhancing disease processes later in life [43]. Endothelin 1, a potent vasoconstrictor and regulator of vascular tone as well as a mediator of inflammation, was shown to be higher in the plasma of young adults with ACEs compared to those without ACEs [3]. Additionally, increased sympathetic activity has been noted in individuals with ACEs [44]. Finally, dysregulation of immune system activity through altered expression or signaling of interleukin 6 (IL-6), tumor necrosis factor (TNF-α), and C-reactive protein can drive vascular stiffness development [45].

Future studies should be devoted to better understand the mechanisms by which ACEs are associated with future CVD risk, which will help in the design of early intervention trials to promote longevity in individuals with early life stress exposure. For example, family-based intervention programs have shown success in reducing poor mental health in those individuals exposed to childhood adversity, but no studies have yet examined the cardiovascular health of patients in such intervention studies. AIx75 could be used as a surrogate outcome in family-based intervention programs for adolescents with ACEs. Given the known association of increased vascular stiffness with poor chronic kidney disease outcomes, the data revealed in this study linking ACEs with increased Alx75 may be key for explaining the association of ACEs with an increase in developing kidney disease [2].

## Supplementary information

Below is the link to the electronic supplementary material.

Graphical Abstract (PPTX 3430 KB)

Supplementary file2 (PDF 599 KB)

## Data Availability

All R scripts necessary to reproduce the statistical analyses and visualizations in this manuscript and the supplemental materials are available upon reasonable request from the corresponding author.
