# Supplementary material for: Adverse childhood experiences are associated with vascular changes in adolescents that are risk factors for future cardiovascular disease
Source: Pediatr Nephrol. 2023 Jan 9;38(7):2155–63. doi: 10.1007/s00467-022-05853-2 (PMC10234926; doi:10.1007/s00467-022-05853-2)
Supplement: Supplementary file 1 — Graphical Abstract (PPTX 3430 KB) [file 467_2022_5853_MOESM1_ESM.pptx]

## Slide 1
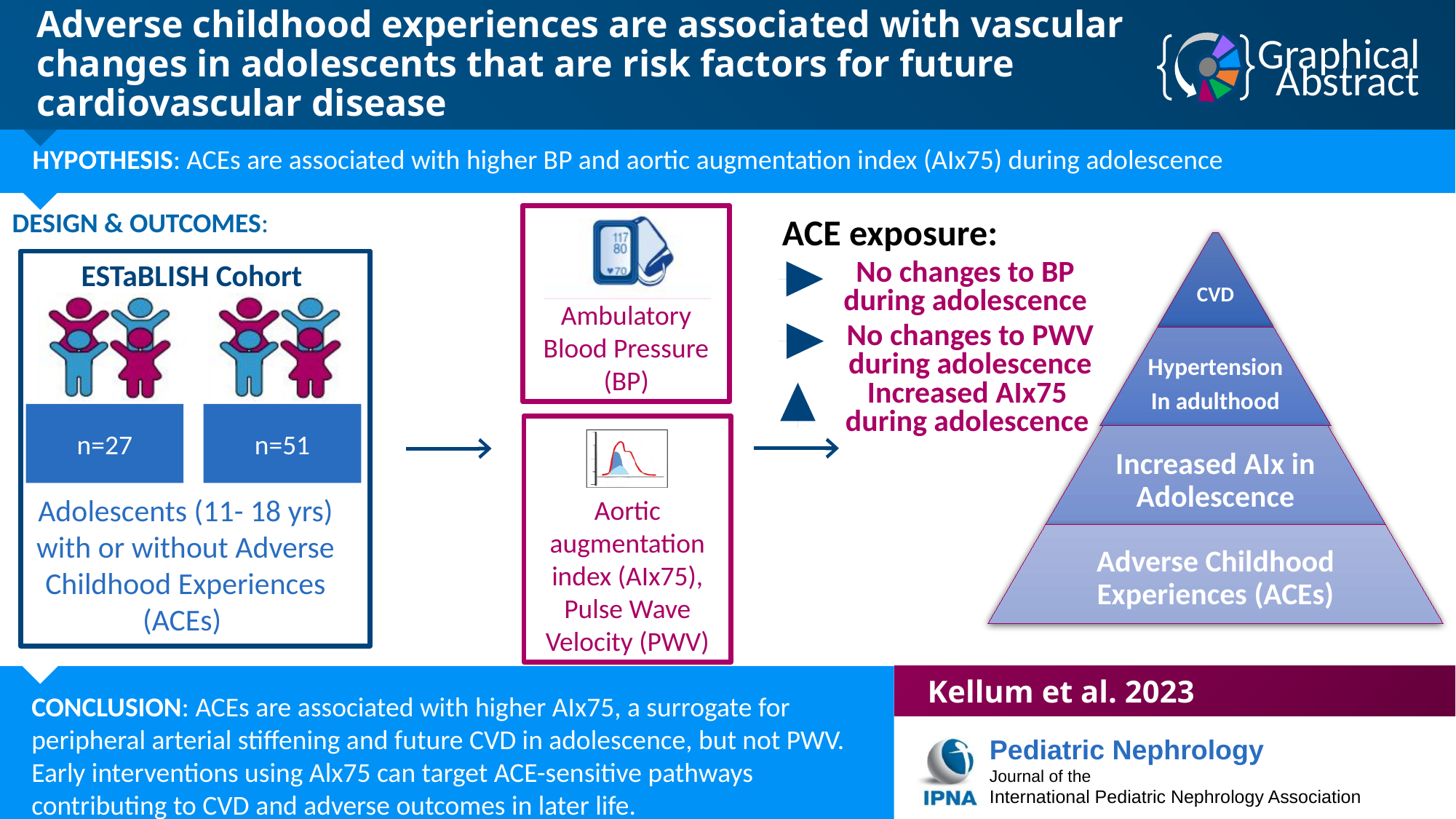

Adverse childhood experiences are associated with vascular changes in adolescents that are risk factors for future cardiovascular disease
HYPOTHESIS: ACEs are associated with higher BP and aortic augmentation index (AIx75) during adolescence
DESIGN & OUTCOMES:
ACE exposure:
Ambulatory Blood Pressure (BP)
ESTaBLISH Cohort
No changes to BP during adolescence
No changes to PWV during adolescence
Increased AIx75 during adolescence
n=51
n=27
Aortic augmentation index (AIx75), Pulse Wave Velocity (PWV)
Adolescents (11- 18 yrs)
with or without Adverse Childhood Experiences (ACEs)
Kellum et al. 2023
CONCLUSION: ACEs are associated with higher AIx75, a surrogate for peripheral arterial stiffening and future CVD in adolescence, but not PWV. Early interventions using Alx75 can target ACE-sensitive pathways contributing to CVD and adverse outcomes in later life.
