# Supplementary material for: Adverse childhood experiences are associated with vascular changes in adolescents that are risk factors for future cardiovascular disease
Source: Pediatr Nephrol. 2023 Jan 9;38(7):2155–63. doi: 10.1007/s00467-022-05853-2 (PMC10234926; doi:10.1007/s00467-022-05853-2)
Supplement: Supplementary file 2 — Supplementary file2 (PDF 599 KB) [file 467_2022_5853_MOESM2_ESM.pdf]

Table S1. Unadjusted effect of adverse childhood experience (ACE) on AIx

	Model 1 (unadjusted)	
Variable	p-value	β coef
ACE (continous)	0.029	0.24
Adjusted R^2	0.046	
F statistic p-value	0.03	
Intercept (p-value)	0.031 (1.00)	

PWV, pulse wave velocity; BMI, body mass index; β coef, standardized beta coefficient

Supplementary Table 1. Linear regression model for ACE exposure measured as a continuous variable as a predictor of AIx75.

Table S2. Unadjusted and adjusted effect of adverse childhood experience (ACE) on PWV

	Model 1 (unadjusted)		Model 2 (adjusted)		Model 3 (adjusted)	
Variable	p-value	β coef	p-value	β coef	p-value	β coef
ACE (0 vs. ≥ 1)	0.85	0.02	0.99	0.0018	0.013	2.87
log(BMI)	-	-	0.003	0.32	<0.001	0.61
Sex	-	-	0.005	0.3	0.003	0.31
ACE (0 vs. ≥ 1) *log(BMI)	-	-	-	-	0.01	-2.92
Adjusted R^2	-0.013		0.17		0.23	
F statistic (p-value)	0.03 (0.85)		6.17 (<0.001)		6.60 (<0.001)	
Intercept (p-value)	5.36 (<0.001)		1.81 (0.099)		-1.14 (0.47)	

PWV, pulse wave velocity; BMI, body mass index; β coef, standardized beta coefficient

Supplementary Table 2. Linear regression model for ACE exposure as a predictor of PWV. ACE exposure does not predict higher PWV (Model 1) and does not predict higher PWV when adjusting for sex and BMI (Model 2). ACE and BMI have a significant interaction (p=0.01).

Supplemental Table 3. Unadjusted and adjusted effect of adverse childhood experience (ACE) on BP

24 Hour

Unadjusted and adjusted effect of adverse childhood experience (ACE) on DBP

	Model 1 (unadjusted)		Model 2 (adjusted)	
Variable	p-value	β coef	p-value	β coef
ACE (0 vs. ≥ 1)	0.15	0.166	0.16	0.16
log(BMI)	-	-	0.21	0.14
Sex (Female)	-	-	0.86	-0.019
ACE (0 vs. ≥ 1) * BMI	-	-	-	-
Adjusted R^2	0.015		0.009875	
F statistic (p-value)	2.16 (0.15)		1.26 (0.30)	
Intercept (p-value)	64.15 (<0.001)		56.34 (<0.001)	

Unadjusted and adjusted effect of adverse childhood experience (ACE) on SBP

	Model 1 (unadjusted)		Model 2 (adjusted)		Model 2 (adjusted)	
Variable			p-value	β coef	p-value	β coef
ACE (0 vs. ≥ 1)	0.87	0.39	0.92	0.009	0.102	1.75
log(BMI)	-	-	<0.001	0.43	<0.001	0.60
Sex (Female)	-	-	0.007	-0.278	0.004	0.29
ACE (0 vs. ≥ 1) * BMI	-	-	-	-	0.10	-1.77
Adjusted R^2	-0.013		0.27		0.26	
F statistic (p-value)	0.03 (0.87)		9.22 (<0.001)		7.76 (<0.001)	
Intercept (p-value)	110.89 (<0.001)		67.47 (<0.001)		43.62 (0.006)	

Unadjusted and adjusted effect of adverse childhood experience (ACE) on MAP

	Model 1 (unadjusted)		Model 2 (adjusted)		Model 2 (adjusted)	
Variable	p-value	β coef	p-value	β coef		
ACE (0 vs. ≥ 1)	0.33	0.11	0.34	1.22	0.11	1.87
log(BMI)	-	-	0.02	5.12	0.006	0.44
Sex (Female)	-	-	0.1	-0.18	0.1	0.2
ACE (0 vs. ≥ 1) * BMI	-	-	-	-	0.13	-1.79
Adjusted R^2	<0.001		0.08		0.1	
F statistic (p-value)	0.95 (0.33)		3.38 (0.02)		3.16 (0.02)	
Intercept (p-value)	80.22 (<0.001)		64.61 (<0.001)		51.76 (<0.001)	

Daytime

Unadjusted and adjusted effect of adverse childhood experience (ACE) on DBP

	Model 1 (unadjusted)		Model 2 (adjusted)	
Variable	p-value	β coef	p-value	β coef
ACE (0 vs. ≥ 1)	0.44	0.089	0.45	0.087
log(BMI)	-	-	0.307	0.12
Sex (Female)	-	-	0.38	-1.15
ACE (0 vs. ≥ 1) * BMI	-	-	-	-
Adjusted R^2	-0.005		-0.006	
F statistic (p-value)	0.60 (0.44)		0.834 (0.48)	
Intercept (p-value)	68.56 (<0.001)		61.525 (<0.001)	

Unadjusted and adjusted effect of adverse childhood experience (ACE) on SBP

	Model 1 (unadjusted)		Model 2 (adjusted)		Model 2 (adjusted)	
Variable	p-value	β coef	p-value	β coef		
ACE (0 vs. ≥ 1)	0.95	-0.007	0.89	-0.14	0.09	1.84
log(BMI)	-	-	<0.001	0.38	0.0002	0.56
Sex (Female)	-	-	0.003	-0.03	0.0017	0.32
ACE (0 vs. ≥ 1) * BMI	-	-	-	-	0.087	-1.88
Adjusted R^2	-0.013		0.22		0.24	
F statistic (p-value)	0.004 (0.95)		8.16 (<0.001)		7.04 (<0.001)	
Intercept (p-value)	115.44 (<0.001)		74.63 (<0.001)		46.98 (0.007)	

Unadjusted and adjusted effect of adverse childhood experience (ACE) on MAP

	Model 1 (unadjusted)		Model 2 (adjusted)		Model 2 (adjusted)	
Variable	p-value	β coef	p-value	β coef	p-value	β coef
ACE (0 vs. ≥ 1)	0.71	0.043	0.72	0.04	0.064	2.19
log(BMI)	-	-	0.04	0.23	0.007	0.44
Sex (Female)	-	-	0.04	-0.23	0.024	0.25
ACE (0 vs. ≥ 1) * BMI	-	-	-	-	0.068	-2.18
Adjusted R^2	-0.011		0.073		0.1	
F statistic (p-value)	0.14 (0.71)		3.03 (0.03)		3.21 (0.02)	
Intercept (p-value)	84.11 (<0.001)		69.44 (<0.001)		51.20 (<0.001)	

Nighttime

Unadjusted and adjusted effect of adverse childhood experience (ACE) on DBP

	Model 1 (unadjusted)		Model 2 (adjusted)	
Variable	p-value	β coef	p-value	β coef
ACE (0 vs. ≥ 1)	0.12	2.24	0.14	2.15
log(BMI)	-	-	0.26	2.70
Sex (Female)	-	-	0.60	0.73
ACE (0 vs. ≥ 1) * BMI	-	-	-	-
Adjusted R^2	0.019		0.013	
F statistic (p-value)	2.46 (0.12)		1.33 (0.27)	
Intercept (p-value)	56.07 (<0.001)		47.00 (<0.001)	

Unadjusted and adjusted effect of adverse childhood experience (ACE) on SBP

	Model 1 (unadjusted)		Model 2 (adjusted)		Model 2 (adjusted)	
Variable	p-value	β coef	p-value	β coef	p-value	β coef
ACE (0 vs. ≥ 1)	0.75	0.037	0.8	0.026	0.29	1.15
log(BMI)	-	-	<0.001	0.442	0.0003	0.55
Sex (Female)	-	-	0.03	-0.22	0.022	0.24
ACE (0 vs. ≥ 1) * BMI	-	-	-	-	0.3	-1.14
Adjusted R^2	-0.012		0.23		0.23	
F statistic (p-value)	0.11 (0.75)		8.46 (<0.001)		6.626 (<0.001)	
Intercept (p-value)	102.93 (<0.001)		52.38 (<0.001)		34.14 (0.057)	

Unadjusted and adjusted effect of adverse childhood experience (ACE) on MAP

	Model 1 (unadjusted)		Model 2 (adjusted)		Model 2 (adjusted)	
Variable	p-value	β coef	p-value	β coef	p-value	β coef
ACE (0 vs. ≥ 1)	0.14	0.169	0.14	2.09	0.31	1.21
log(BMI)	-	-	0.015	5.71	0.021	0.37
Sex (Female)	-	-	0.2	-1.71	0.17	0.15
ACE (0 vs. ≥ 1) * BMI	-	-	-	-	0.37	-1.06
Adjusted R^2	0.016		0.089		0.087	
F statistic (p-value)	2.25 (0.14)		3.51 (0.019)		2.82 (0.03)	
Intercept (p-value)	72.70 (<0.001)		55.04 (<0.001)		46.17 (<0.001)	

Supplementary Table 3. Linear regression models for ACE exposure as a predictor of BP. ACE exposure does not predict higher 24h, daytime, or nighttime DBP/SBP/MAP.

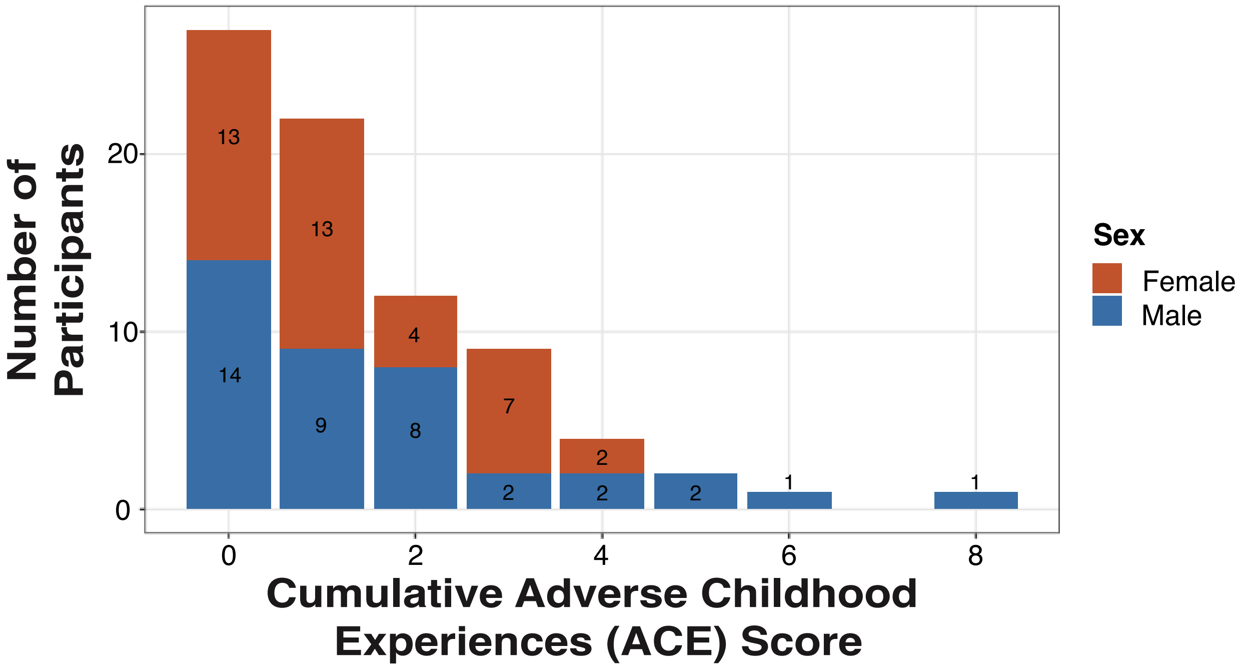

Supplementary Figure 1. Visual representation of the distribution of ACEs in the study cohort. In analysis, ACEs of 1 or more were grouped together.

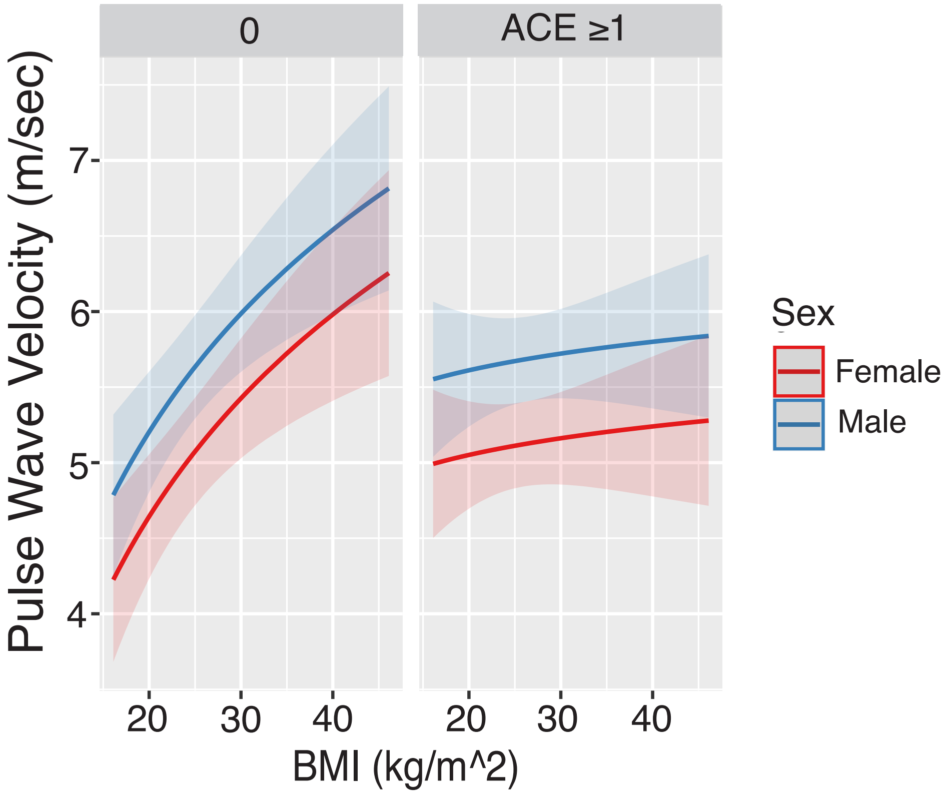

Supplementary Figure 2. Visual representation of the linear regression model of PWV with a BMI and ACE exposure interaction while adjusting for sex.(Table S2: Model 3). The interaction of ACE exposure and BMI is a significant predictor of PWV (beta -2.92; p =0.01).The effect of BMI on PWV is less positive in the ACE group compared to the No ACE group.
